# A standard protocol for the analysis of postmortem muscle protein degradation: process optimization and considerations for the application in forensic PMI estimation

**DOI:** 10.1007/s00414-022-02849-3

**Published:** 2022-06-17

**Authors:** Stefan Pittner, Veronika Merold, Sven Anders, Larissa Lohner, Jens Amendt, Miriam Klinger, Roland Hausmann, Steffen Kissling, Fabio Monticelli, Janine Geissenberger, Angela Zissler, Peter Steinbacher

**Affiliations:** 1 Department of Forensic Medicine, Paris-Lodron University of Salzburg, Salzburg, Austria; 2 Department of Environment and Biodiversity, Paris-Lodron University of Salzburg, Salzburg, Austria; 3 Department of Legal Medicine, University Medical Center Hamburg-Eppendorf, Hamburg, Germany; 4 Institute of Legal Medicine, Goethe-University, Frankfurt, Frankfurt, Germany; 5 Institute of Legal Medicine, Cantonal Hospital St, Gallen, St. Gallen, Switzerland

**Keywords:** Protocol, Muscle, Protein, Degradation, PMI

## Abstract

The analysis of postmortem protein degradation has become of large interest for the estimation of the postmortem interval (PMI). Although several techniques have been published in recent years, protein degradation-based techniques still largely did not exceed basic research stages. Reasons include impractical and complex sampling procedures, as well as highly variable protocols in the literature, making it difficult to compare results. Following a three-step procedure, this study aimed to establish an easily replicable standardized procedure for sampling and processing, and further investigated the reliability and limitations for routine application. Initially, sampling and processing were optimized using a rat animal model. In a second step, the possible influences of sample handling and storage on postmortem protein degradation dynamics were assessed on a specifically developed human extracorporeal degradation model. Finally, the practical application was simulated by the collection of tissue in three European forensic institutes and an international transfer to our forensic laboratory, where the samples were processed and analyzed according to the established protocol.

**Supplementary information:**

The online version contains supplementary material available at 10.1007/s00414-022-02849-3.

## Introduction

Analysis of postmortem tissue degradation has become of particular interest in recent years for determining the postmortem interval (PMI) [1]. In particular, analysis of protein decomposition has proven its potential to contribute to the methodic spectrum in postmortem stages where other methods fail to obtain reliable data [1]. The range of approaches investigating protein degradation is wide and differs in methods [2–5], tissues [6–8], and target proteins [1]. However, until today, these techniques are rarely applied in routine investigations [9]. Most of what is published as a “novel method for PMI estimation” does in fact not exceed very basic research stages due to several reasons: (i) impractical protocols for routine application, (ii) vast heterogeneity of reference literature regarding techniques and protocols and, thus, restricted comparability of results, and (iii) limited understanding of the methodic boundaries, such as influencing factors and exclusion criteria.

(i) An important task in protein degradation-based PMI estimation is to stop the degradation process of tissue at the time of sampling, which is often best achieved by snap freezing and storing the samples in liquid nitrogen [1, 5, 9, 10], if structural integrity can be disregarded. However, because this procedure is impractical for routine application (immediate provision of liquid nitrogen, high acquisition costs, complicated transport, etc.), alternatives are required.
(ii) Apart from the difficulty to transfer data from animal models to humans [11], from one organ, tissue, or body part to another [7, 12, 13], or from one analysis technique to another, practical application can be challenging due to inherent variations of techniques. While (basic) research can be carried out under standardized conditions, these exact conditions rarely occur in routine work. Additionally, complex and time-consuming sample preparations are inconvertible in the morgue and in the field [14].
(iii) As a metabolic process, proteolysis underlies several influencing factors including individual and environmental properties, as well as circumstances of death [15]. Additionally, the effects are not limited to in situ tissue, but likewise affect a sample once it has been collected [16–18], thus potentially biasing the interpretation of the analysis.

The present study aims to investigate the influences of sample collection, processing, and storage on the outcome of protein degradation analysis in skeletal muscle tissue for PMI estimation, to establish a reliable standard protocol for routine practice and to investigate its methodological limitations. Following a three-step procedure, a rat animal model was developed to investigate the impact of various sample preparation techniques on the outcome quality of protein degradation analysis by Western blotting in an initial step. Applying the obtained protocols, a human extracorporeal protein degradation model was deployed using muscle tissue blocks from five autopsy cases, to test the robustness of the protocols towards sample transfer and storage. Ultimately, application was tested in three forensic institutes in Germany and Switzerland. Obtained samples were transferred to Austria for analysis to test the chain of custody and establish a test routine.

## Material and methods

### Animal model

**Fig. 1:**
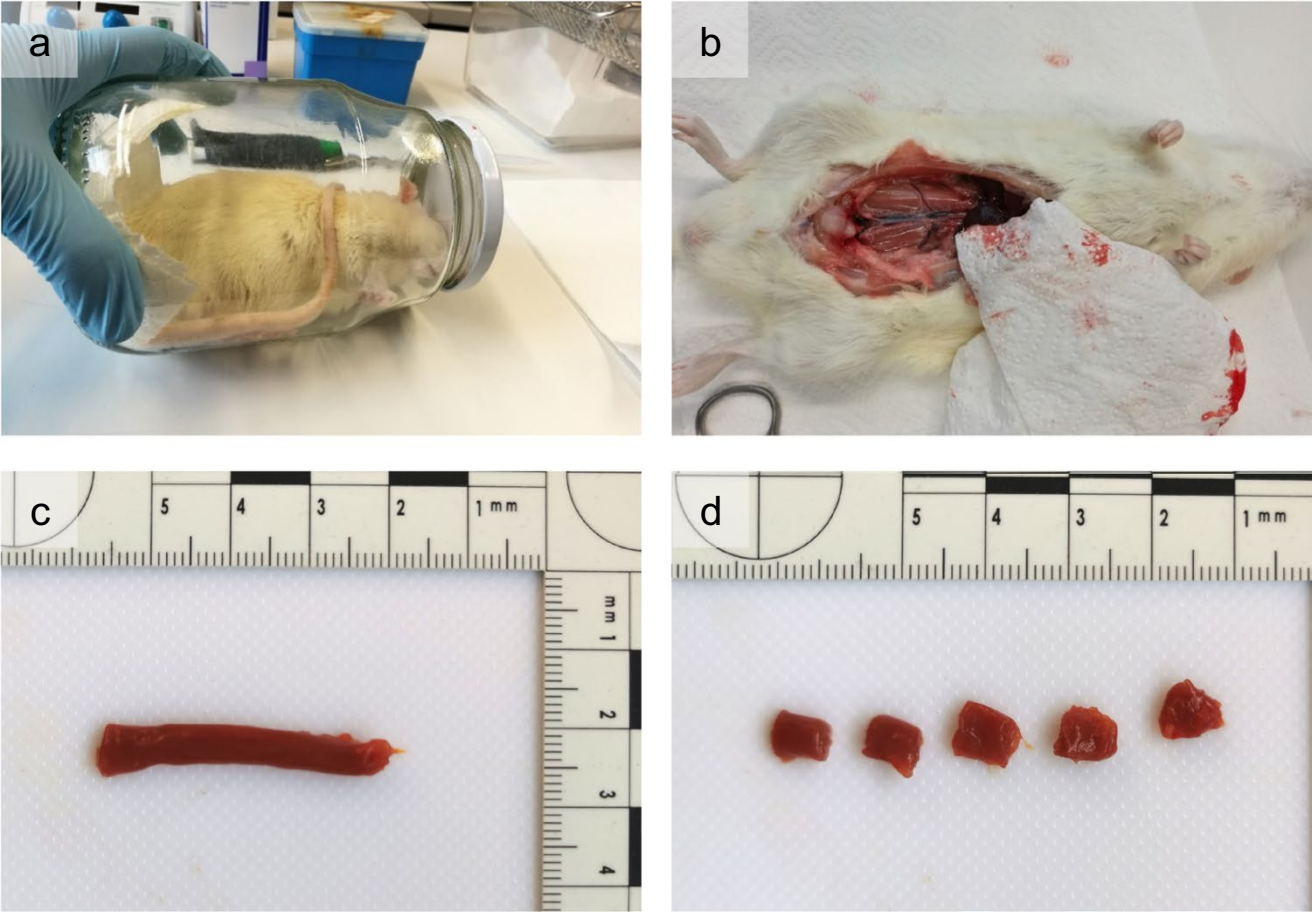
Sample collection for the animal model. **a** Rat anesthesia with a piece of cloth containing a lethal dose of isoflurane. **b–c** Abdomen was opened with a scalpel, GI tract removed, and *M. psoas major* extracted. **d** Subdivision of *M. psoas major* into five individual samples of approximately 5 × 5 × 5 mm (approx. 100 mg)

Three adult male Sprague Dawley rats were used to model postmortem protein degradation. The animals were carefully put into a glass jar containing a piece of cloth with 1 ml of isoflurane. The jar was then covered with a towel to provide a darker environment and calm the animals down. Once the rats were in deep anesthesia, they were killed by cervical dislocation and two specimens were immediately transferred into a climate chamber at controlled environmental conditions (20 °C, 50% RH) and stored for 24 (day 1) and 72 h (day 3). The third rat was immediately dissected and the left *M. psoas major* removed. This muscle was chosen for its shape and size so that it could subsequently be easily divided into five equal sized subsamples along its longitudinal axis (Fig. 1).

The first subsample was snap frozen in liquid nitrogen (*cryo*); a second sample was directly transferred into a vial containing 1 ml of extraction buffer (RIPA buffer (SIGMA) and protease inhibitor cocktail (ROCHE)) without further subdivision (*no subdivision*). To increase the surface area and thus improve buffer infiltration, the remaining samples were further subdivided into smaller pieces (< 1 mm in one direction) and transferred to vials containing 0.5 ml extraction buffer (*0.5 ml buffer vol*), 1 ml extraction buffer (*standard*), and 2 ml extraction buffer (*2 ml buffer vol*).

For protein extraction, all samples in RIPA buffer were homogenized using an Ultra Turrax disperser (IKA Werke GmbH & CO. KG) after 30 min of incubation at room temperature. Samples stored in liquid nitrogen were homogenized by cryogenic grinding and subsequently added to 10 × vol/wt of extraction buffer. For secondary homogenization, all samples were treated with ultrasound (2 × 100 Ws/sample) and subsequently centrifuged at 1000 × g for 10 min. The supernatants were transferred and stored at − 20 °C until further use. Protein concentrations were measured by using Pierce BCA-Assay Kit (Thermo Fisher Scientific Inc.).

### SDS-PAGE and Western blotting

SDS-PAGE was performed according to Laemmli [19] with some adaptions. A detailed protocol is available as supplementary document (Supplementary File 1).

Protein band intensities were measured using ImageJ software (ImageJ 1.45 s, Java 1.6.0_20). Signals of the native bands with an intensity < 1% were considered background and thus no band. Alterations of band patterns, such as the disappearance of a native band or appearance of additional bands, were considered degradation events. For the depiction in the included figures, lanes were cropped, pasted, and adjusted for brightness and contrast.

### Human extracorporeal degradation model

To optimize the applicability of the established protocols for routine use, a human extracorporeal protein degradation model was developed. Therefore, samples from five autopsy cases from the Dept. of Forensic Medicine in Salzburg were collected. The following inclusion (i) and exclusion (ii) factors were considered: (i) age between 18 and 80 years, BMI between 18.5 and 30 (neither underweight, nor obese), PMI < 48 h (including cooling time at the facility); (ii) thigh trauma (e.g., fracture, open wound, hematoma), known muscle associated disease (e.g., dystrophy), possible atrophy due to immobility (e.g., bedriddenness, paralysis, cast), circumstances of death possibly influencing protein degradation (e.g., burning, freezing). Table 1 summarizes the data of the included cases.

**Table 1:** Summary of demographic and death-related data of the included cases. *BMI*, body mass index; *PMI*, postmortem interval. Total PMI includes cooling time

Case no	Sex	Age [y]	BMI	Cause of death	Total PMI [h]	Cooling time at 5 °C [h]
1	f	23	25.8	Internal bleeding (stabbing to neck and chest)	39	9
2	f	63	26.1	Polytrauma (traffic accident)	30	26
3	m	48	25.7	Intoxication	27	21
4	f	51	23.3	Internal bleeding (multiple gunshot wounds)	15	6
5	f	77	29.2	Internal bleeding (multiple gunshot wounds)	15	6

### Sample collection and processing

**Fig. 2:**
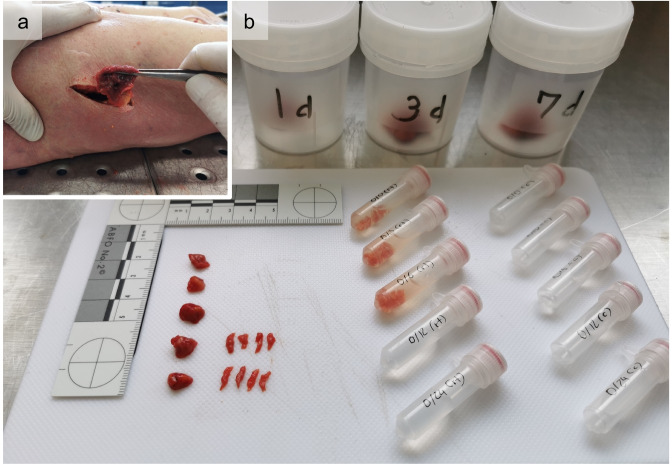
Sample collection and division. **a** Incision in the lateral thigh and extraction of a muscle tissue block. **b** Sample subdivision: The sample block was divided into four smaller pieces, one for each day of the extracorporeal protein degradation model (cups in the background). On each time point, subsamples (approx. 100 mg) were obtained for protein analysis (bottom left), cut into pieces < 1 mm in one direction (bottom half left), and transferred into vials containing extraction buffer (bottom right)

During autopsy, a 5 cm incision was made in the center of the lateral thigh. Thereby, skin, fat tissue, and muscle fascia were opened. Muscle samples (*M. vastus lateralis*) of approximately 4 × 4 × 4 cm were collected from medium depth (3–8 cm, depending on constitution, approximately half the distance to the femur) and subdivided into four smaller pieces. It was taken care that four equal portions in size and shape were produced when dividing the tissue blocks. Fat, larger vessels and connective tissue were removed, leaving only muscle tissue. One of the subsamples was processed immediately as described below, while the three remaining subsamples were stored in closed plastic containers at room temperature for 1, 3 and 7 days to model extracorporeal protein degradation and compare the progression of possible degradation events. At each time point, one subsample was further divided into 10 smaller pieces of about 5 × 5 × 5 mm (approx. 100 mg). These samples were additionally cut to smaller pieces (< 1 mm in one direction) with a scalpel to obtain larger surface areas and thus optimize contact with the extraction buffer. The cut pieces were then transferred into 10 vial tubes containing 1 ml of extraction buffer (RIPA buffer (SIGMA) and protease inhibitor cocktail (ROCHE)) and incubated at room temperature for 30 min (Fig. 2).

Five tubes were stored at room temperature (20 °C) and five tubes were frozen at − 20 °C. The samples stored at room temperature were further processed after a storage time of 0, 3, 6, 12, and 24 h. Frozen samples were thawed after one week and processed after another 0, 3, 6, 12, and 24 h storage time at 4 °C. Table 2 provides on overview of all collected samples per case. Sample processing and protein analysis was performed as described above (animal model).

**Table 2:** Overview of the samples collected per case. The label *a/b(x)* describes the storage conditions of each subsample. *a* indicates the duration of storage of the fresh (unprocessed) sample block (extracorporeal PMI) in days. *b* indicates the storage time (in hours) of the processed sample before further analysis. *(x)* indicates the storage regime (*rt*, room temperature: 20 °C; *c*, cold: 4 °C). 0 h room temperature samples were considered controls and used to evaluate the extracorporeal degradation model

		b. sample storage									
		room temperature [20 °C]					cold [4 °C]				
		0 h (control)	3 h	6 h	12 h	24 h	0 h	3 h	6 h	12 h	24 h
a. tissue storage at room temperature [20 °C]	**0 d**	*0/0(rt)*	*0/3(rt)*	*0/6(rt)*	*0/12(rt)*	*0/24(rt)*	*0/0(c)*	*0/3(c)*	*0/6(c)*	*0/12(c)*	*0/24(c)*
	**1 d**	*1/0(rt)*	*1/3(rt)*	*1/6(rt)*	*1/12(rt)*	*1/24(rt)*	*1/0(c)*	*1/3(c)*	*1/6(c)*	*1/12(c)*	*1/24(c)*
	**3 d**	*3/0(rt)*	*3/3(rt)*	*3/6(rt)*	*3/12(rt)*	*3/24(rt)*	*3/0(c)*	*3/3(c)*	*3/6(c)*	*3/12(c)*	*3/24(c)*
	**7 d**	*7/0(rt)*	*7/3(rt)*	*7/6(rt)*	*7/12(rt)*	*7/24(rt)*	*7/0(c)*	*7/3(c)*	*7/6(c)*	*7/12(c)*	*7/24(c)*

### External cases

To ultimately test the applicability of the established protocol, additional cases from Institutes of Legal Medicine in Germany (Frankfurt/Main and Hamburg) and Switzerland (St. Gallen) were included. Case information is summarized in Table 3.

**Table 3:** Summary of demographic and death related data of the included cases from Institutes of Legal Medicine in Germany and Switzerland. *BMI*, body mass index; *PMI*, postmortem interval. Total PMI includes cooling time. *BMI not available (no accurate height determined due to severe injuries)

Case no	Sex	Age [y]	BMI	Cause of death	Total PMI [h]	Cooling time at 5 °C [h]
FRA1	M	43	27.8	Suffocation	28	20
FRA2	M	63	36.2	Myocardial infarction	100	~ 80
SGA1	F	23	n.a.*	Polytrauma (train assisted suicide)	5	0
SGA2	M	26	34.3	Polytrauma (traffic accident)	19	6
HAM1	M	51	25.1	Pneumonia	34	22
HAM2	M	18	20.7	Intracranial hemorrhage	194	186

A detailed description of the sampling procedure is available as supplementary document (Supplementary File 1). Frozen samples were packed into Styrofoam boxes equipped with cool packs and insulating layers and transferred to Salzburg/Austria for further processing and analysis of protein degradation.

## Results

### Animal model

All samples were processed according to the respective protocols. Both standard procedures (“cryo” and “standard”) produced almost identical and very decent, clear bands with negligible background staining. Vinculin depicted a native band at approximately 117 kDa on day 1, day 2, and day 3 (with a tendency to decrease in intensity over time) and an additional band above 117 kDa (considered to be the splice-variant meta-vinculin [20]) on day 0. On day 1 and day 3, additional bands < 84 kDa were detected (degradation products). Exclusively, the samples collected at day 0 showed a single α-tubulin band at approximately 55 kDa. All samples depicted the characteristic double band pattern of tropomyosin at approximately 37 kDa.

**Fig. 3:**
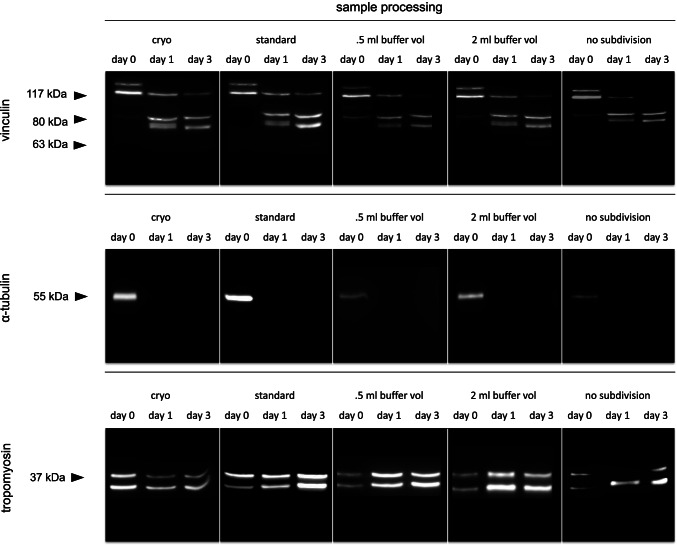
Western blot analysis of rat muscle tissue samples with differences in sample preparation. Cryo: Samples were snap frozen in liquid nitrogen and homogenized by cryogenic grinding. Standard: Samples were cut into small pieces, 1 ml of extraction buffer added and homogenized using a tissue disperser. 0.5 and 2 ml buffer vol: Buffer volume was halved, respectively doubled. No subdivision: Samples were not cut into smaller pieces prior to homogenization

The measured overall protein concentration was low in *0.5 ml buffer vol* samples and in *2 ml buffe*r *vol* samples, and even lower in *no subdivision* samples. The concentrations were sufficient for further analysis but bands were less distinct in all proteins analyzed. In the 0.5* ml buffer vol* samples and in *2 ml buffe*r *vol* samples the native vinculin band of the day 3 sample was below the detection threshold and could have produced a wrong-negative result. Samples not subdivided prior to the transfer into the buffer vials (*no subdivision*) obtained the poorest band quality. All present bands were faint and again the native vinculin band of the day 3 sample as well as a tropomyosin band of a day 1 sample was absent (Fig. 3).

### Human extracorporeal degradation model

During extracorporeal sample storage, no visual changes such as drying and discoloration could be documented for any of the samples. Further cutting of the samples to enlarge the surface area resulted in optimal sample infiltration of extraction buffer solution and thus decent overall protein concentrations sufficient to produce distinct protein bands.

**Fig. 4:**
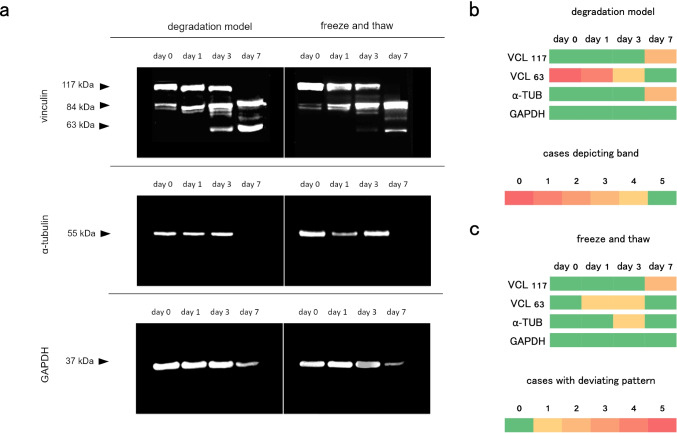
**a** Western blot images of postmortem protein degradation patterns. *Left:* Developed protein degradation model at 0, 1, 3, and 7 days of extracorporeal storage at 20 °C. Vanishing native bands (117 kDa vinculin band and 55 kDa α-tubulin band at 7 days) and appearing degradation products (63 kDa vinculin band) were considered degradation events. *Right:* representative protein degradation patterns after freezing (for a week) and thawing. **b** Summary of protein degradation patterns in all 5 analyzed cases. Presence (green) and absence (red) of protein bands in the protein degradation model. **c** Number of samples deviating from the pattern of the degradation model after a freeze and thaw cycle

In a first step, it was tested whether the established extracorporeal degradation model produced reliable postmortem degradation patterns that could be used to investigate the effect of changing parameters (freeze and thaw as well as storage condition and duration). Therefore, the control samples (0 h room temperature storage) of the five included cases were analyzed. Samples of all five cases depicted a native band of vinculin at approximately 117 kDa and a degradation product at 84 kDa. At day 1 post sampling, one case (case 5) depicted an additional degradation product at 63 kDa, while all other cases did not show a change in their protein pattern. On day 3, samples of four cases (all but case 3) depicted all three bands. On day 7, samples of all cases depicted the 63 kDa vinculin degradation product, and in two samples (cases 2 and 5), the native vinculin band could no longer be detected. A single α-tubulin band at approximately 55 kDa was detected in samples of all cases on days 0, 1, and 3 of extracorporeal storage. On day 7, this band was no longer present in samples of two cases (cases 4 and 5). A single GAPDH band at approximately 25 kDa was detected in samples of all cases at all time points (Fig. 4).

### Freeze and thaw

Comparing the outcome of protein analyses after an initial freezing period of 1 week revealed only minor changes of the protein degradation patterns. The native vinculin band in samples of cases 1 and 4 was no longer be detected on day 7. In samples of case 4, the vinculin degradation product was already found on day 1, whereas case 2 lacked this band on day 3, and the α-tubulin band in the case 5 sample was already undetectable on day 3 (Fig. 4).

### Storage conditions and duration

**Fig. 5:**
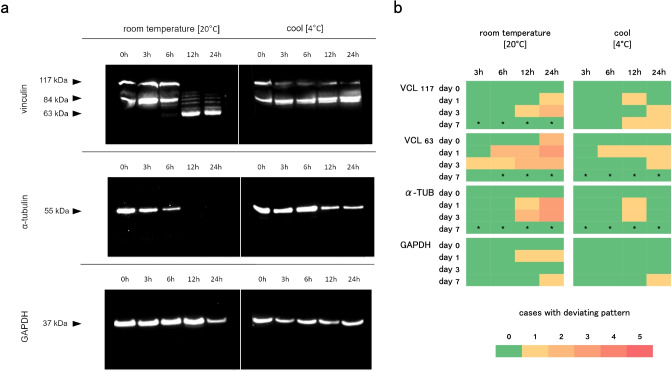
**a** Representative Western blots of samples stored for several hours in two different environments. Only samples from day 3 during extracorporeal storage are shown (these underwent changes most often). **b** Number of cases with alternated protein patterns due to sample storage in two different environments (compared to 0 h). *Asterisks indicate that no single protein band was detected in this group, limiting the validity of comparison

Upon room temperature storage, no deviations to the 0 h storage (control) samples were detected in any of the native bands after 3 and 6 h of storage (no wrong negatives, on behalf of vanishing native bands). After 12 h, samples from 2 of 5 cases showed deviations to the control pattern, and after 24 h deviations were observed in samples of 3 cases in all of the analyzed proteins. Although only in a single case, additional vinculin degradation products were detected after 3 h of storage at room temperature (Fig. 5).

Retention of the protein decomposition status was clearly improved in cold environment. Only samples of individual cases (1 of 5) showed alternated native protein patterns compared to the 0 h storage control. This was the case at 12 h and 24 h of storage. Also in cold storage conditions, samples of a single case showed and additional vinculin degradation product after 6 h (Fig. 5).

### External cases

As for the transport of samples between institutes, it was noted that all samples arrived within 24 h in still cooled packages (< 10 °C), but not all of the samples remained frozen. The logistic effort and timing on this regard was underestimated.

No differences were observed on behalf of homogenization and processing of the muscle samples from the institutes in Germany and Switzerland. Protein concentration measurements revealed sufficient overall protein levels in all analyzed samples.

**Fig. 6:**
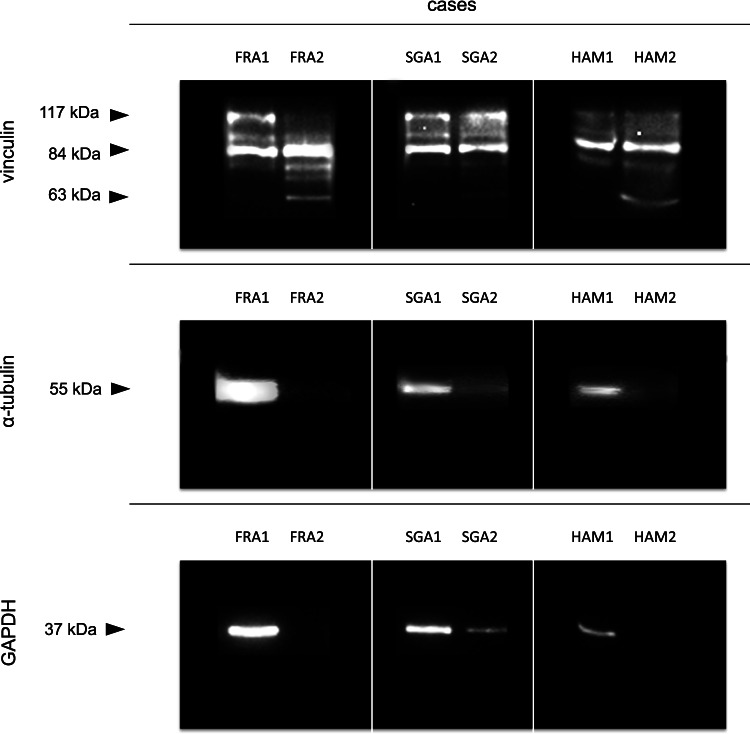
Western blot analyses of the samples collected from cases from forensic institutes in Frankfurt/Main (FRA1-2), St. Gallen (SGA1-2), and Hamburg (HAM1-2)

All samples of cases from St. Gallen (SGA1-2) and Hamburg (HAM1-2) and the first sample from Frankfurt (FRA1) depicted the native vinculin band at 117 kDa, as well as the 84 kDa degradation product. FRA2 did not show a native vinculin band, but instead the familiar 84 and 63 kDa degradation products, the latter also being present in HAM2. The native α-tubulin band was detected in the three first cases from St. Gallen (SGA1), Frankfurt (FRA1), and Hamburg (HAM1) and as a slight band below the intensity threshold of 1% in SGA2. Similarly, GAPDH was detected in the first cases from all three institutes (FRA1, SGA1, and HAM1), as well as in SGA2 (Fig. 6).

## Discussion

This study succeeded to develop a standard protocol for a protein degradation-based PMI method providing a basis for standardized sampling, processing, and analysis. In a three-step process, including the establishment of two degradation models (animal and human) and the testing of the protocol on postmortem tissue sampled from three European forensic institutes, important implications were obtained regarding the outcome of different sample preparation techniques on Western blot band quality and reliability. The developed standard protocol, using an extraction buffer, performed very well in comparison to physical fixation in liquid nitrogen. Distinct bands with limited background were obtained, depicting identical degradation patterns, such as a loss of native bands (α-tubulin) and appearance of degradation products (vinculin). This is in accordance with previous studies in animals and humans [14, 21, 22]. However, it should be noted that analysis of the protein desmin was hindered using this protocol in a previous study [14], as no native bands were detected. This underlines the importance of comparative analyses.

### Animal and human models

We established reliable models for testing protocol alterations such as buffer volume adaptations and processing of the sample, freeze and thaw cycles, and storage conditions and durations. With the established human extracorporeal degradation model, we confirmed the results of previous studies [16–18], demonstrating that protein degradation is not limited to in situ conditions, but progresses in a predictable and consistent manner once the organ/tissue has been sampled. Here, human protein degradation was replicated in five cases, with only minor deviations. However, it should be avoided to conclude from the established extracorporeal degradation model to the dynamics of postmortem protein decomposition in humans in situ. A direct comparison of intra- and extracorporeal decomposition in future studies will allow important insights in this matter. Similarly, in the animal model, conclusions about the timing of the observed postmortem degradation patterns would be invalid due to the small sample size.

### Buffer volume adaptation and optimal processing of the samples

Interestingly, adjustments to the buffer volume in which the 100 mg samples were processed led to worse results, regardless of whether more or less volume was used. However, what appears counter-intuitive on first sight can be explained when the processing procedure is considered. Halved buffer volume led to difficulties during homogenization, as the volume was insufficient to contain the entirety of the sample and proportionally retained a larger amount of fat, connective tissue and cell debris in the sample, contaminating the protein solution. In contrast to the assumption, the samples did not show any irregular bands and no increased background staining. Instead, band intensities substantially decreased. Contrary, a double buffer volume simply led to a proportionally smaller overall protein concentration in the sample and thus fainter bands and increased vulnerability for errors in all subsequent steps.

In terms of sample infiltration, further subdividing the samples into smaller pieces improved band quality compared to uncut samples. The penetration of tissue by the buffer solution is based on diffusion and requires time. Depending on storage temperature, osmolality, hydrophobic interactions, etc., infiltration rates vary from one to several hours per millimeter [23, 24]. An incubation time of 30 min may therefore have been insufficient to appropriately infiltrate a 5 × 5 × 5 mm sample. Because immediate stopping of autolysis and further degradation of the dissected sample are of high importance in PMI estimation, extension of the incubation time should be avoided. Therefore, a subdivision of the extracted samples to a size of < 1 mm in one direction is recommended to keep the incubation period as short and effective as possible.

### Freeze and thaw cycles and storage conditions

The investigation of whether freezing (and thawing) of unprocessed samples can alter the outcome of protein analysis, resulted in deviations of five bands (6.25% of all analyzed bands). Although this small ratio is more likely explained by dispersion rather than by a systemic effect (particularly since both, “advanced” and “delayed” degradation patterns were amongst the deviations), it is recommended to carefully observe this issue in the future. It is known that freezing and thawing can have a dramatic effect on histological microstructure [25], but there are contradictory statements regarding changes at protein level [26, 27]. No effects were observed in the proteins investigated in this study and in previous degradation models [22]. Nevertheless, unnecessary freezing and thawing cycles should be avoided if possible, e.g., during the transfer of sample material.

Contrary, it was shown that sample storage and transfer in a non-frozen state potentially alters the protein degradation process. Although the extraction buffer contains a cocktail of protease inhibitors to limit post-sampling degradation, storage in room temperature conditions for several hours resulted in 31 band alterations (9.69% of all analyzed bands) in comparison to the (0 h storage) control samples. In detail these were 1 alteration (1.25%), and 3 (3.75%), 9 (11.25%), and 18 (22.50%) alterations in the tested storage times of 3, 6, 12, and 24 h respectively. As expected, storage in cold conditions (4 °C) clearly reduced this effect. In total, 11 alterations in comparison to the (0 h storage) control samples were counted (3.44%). These were 0 (0%), 1 (1.25%), 5 (6.25%), and 5 (6.25%) alterations in the tested storage times of 3, 6, 12, and 24 h respectively. For both temperatures, there is a clear trend that longer non-frozen storage times lead to more deviations. Although short non-frozen storage periods seem to have a minor effect, keeping the samples frozen at all time (except during processing and analysis) is clearly recommended. In addition, samples stored at room temperature for longer than the buffer infiltration period should be excluded from analysis for PMI estimation. If unavoidable, storage in cool conditions (4 °C) for short periods (e.g., during sample transportation) should be preferred over room temperature.

### Standard protocol testing

The protocol was adapted according to the above findings and tested for applicability in three forensic institutes in Germany (Hamburg and Frankfurt/Main) and Switzerland (St. Gallen). The required material and equipment is minimal, with only (i) vials containing 1 ml of extraction buffer, (ii) common autopsy instruments (scalpel and forceps), and (iii) the possibility to store the collected samples at − 20 °C. The used biopsy needles are optional, but improve standardization and facilitate sampling.

Although the recommended restrictions were not exceeded, a future transfer of the samples should be improved using dry ice as a cooling medium instead of cool bags to keep the samples frozen for the whole transport period. Sample processing and analysis of protein degradation worked without any problems and, although over-interpretation should be avoided, the obtained protein patterns reflected the respective PMIs well, both in comparison with the established animal model, the extracorporeal degradation model, and literature data [13, 21, 22]. At this point, the available data is insufficient to compare extracorporeal protein degradation to actual (in situ) postmortem decomposition. However, this work provides the basis for according future studies.

The institutional collaboration will be continued in the future to expand the pool of samples and to mutually develop a reliable reference database for PMI estimation by analysis of postmortem protein degradation.

## Supplementary information

Below is the link to the electronic supplementary material.

Supplementary file1 (PDF 18983 KB)
