# Supplementary material for: A standard protocol for the analysis of postmortem muscle protein degradation: process optimization and considerations for the application in forensic PMI estimation
Source: Int J Legal Med. 2022 Jun 17;136(6):1913–23. doi: 10.1007/s00414-022-02849-3 (PMC9576658; doi:10.1007/s00414-022-02849-3)
Supplement: Supplementary file 1 — Supplementary file1 (PDF 18983 KB) [file 414_2022_2849_MOESM1_ESM.pdf]

Protocol for standardized preparation of skeletal muscle tissue and analysis of postmortem protein degradation

1) Material:

- vial tubes with 0.75ml RIPA extraction buffer + protease inhibitor-cocktail (store at 4°C)
- scalpel blade, or if available biopsy needle (5mm diameter)
- forceps
- if available: tissue disperser (e.g. ultra turrax)

2) Sampling procedure:

- Wear examination gloves
- Make a 1-2 cm incision in the center of the lateral thigh muscle. Thereby open skin, fat tissue and muscle fascia.

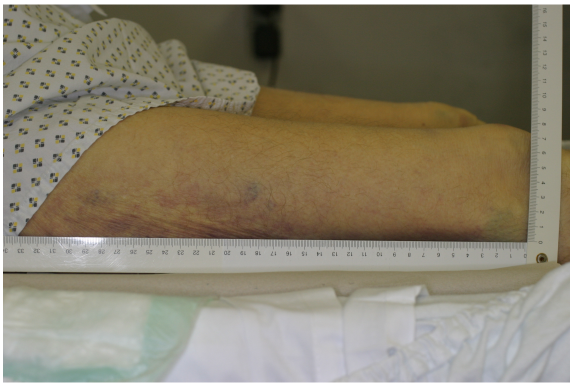

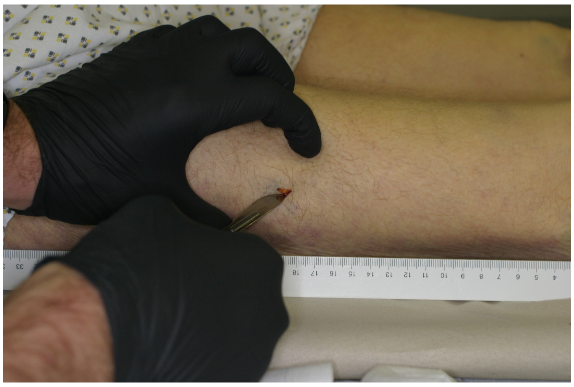

- Excise muscle samples (approx. 5×5×5 mm) from medium depth (3-8 cm, depending on constitution, approximately half the distance to the femur) and subdivide the sample to smaller pieces.

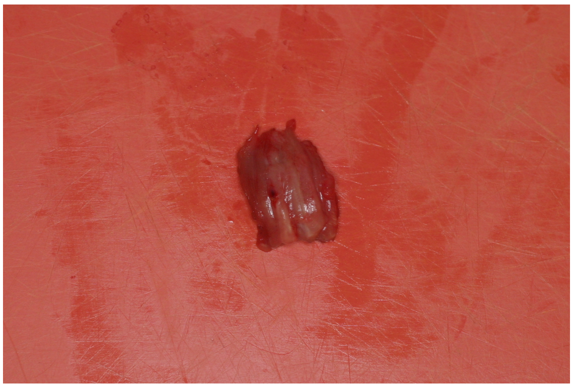

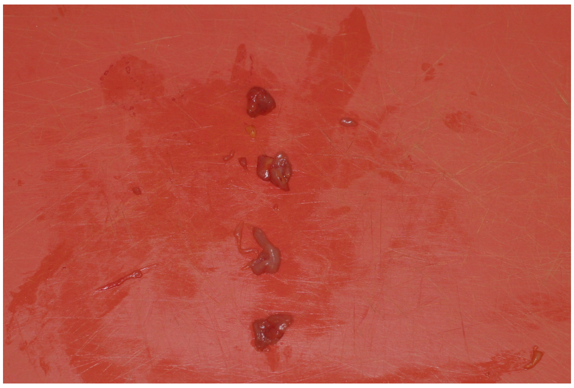

- Alternatively obtain several biopsy samples from equal depth (described before).

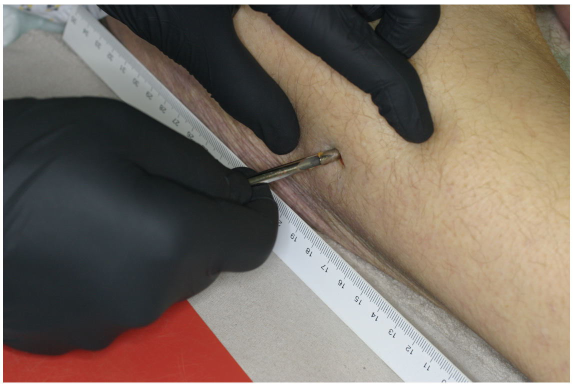 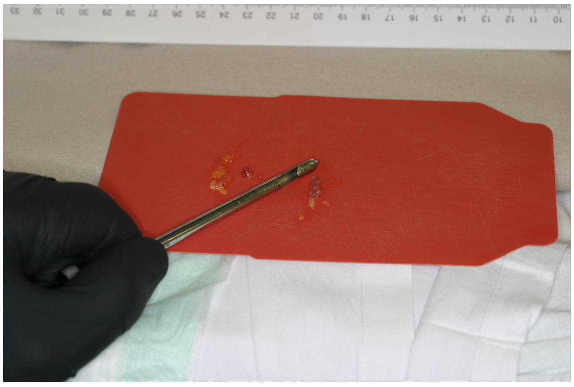

- Transfer the obtained samples into the vial tubes, shake briefly to prevent them from sticking to the wall and store them upright ("standing") at -20°C.

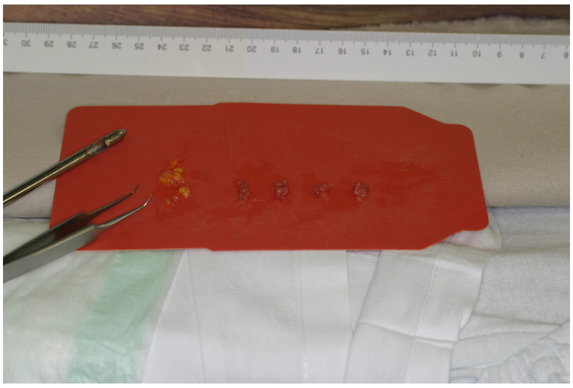 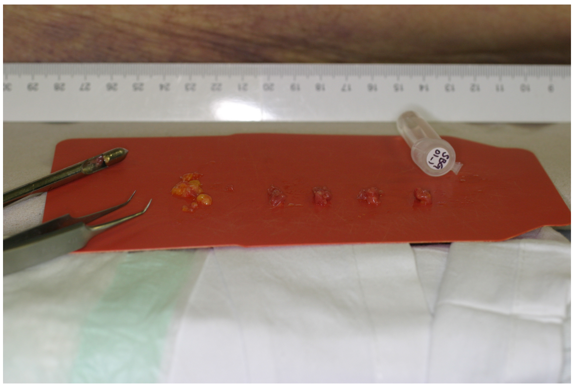

- If available, homogenize the samples prior to storage using a tissue disperser.

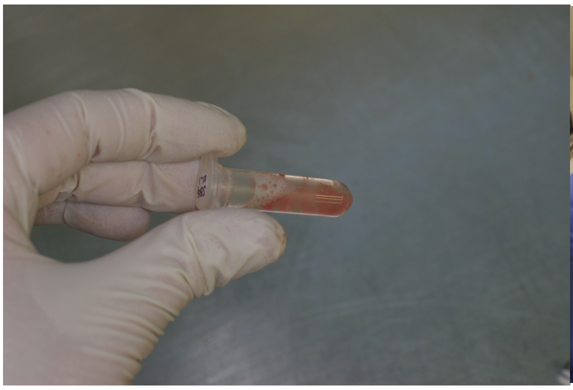 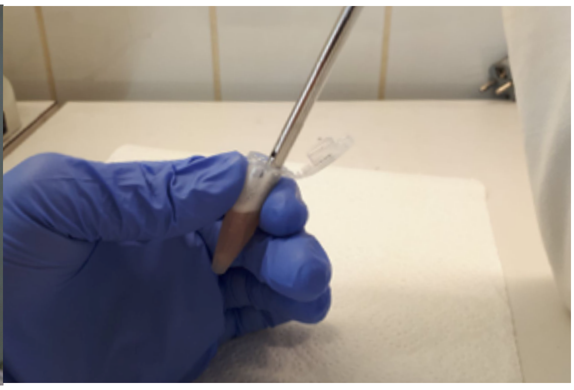

3) SDS PAGE and Western Blotting:

Electrophoreses is run on 10 % polyacrylamide resolving gels (acrylamide/N,N'-bismethylene acrylamide = 37.5:1, 0.1 % SDS, 0.05 % TEMED, 0.05 % APS, 375 mM Tris HCl, pH 8.8) and 5 % stacking gels (acrylamide/N,N'-bismethylene acrylamide = 37.5:1, 0.1 % SDS, 0.125 % TEMED, 0.075 % APS, 125 mM Tris HCl, pH 6.8). 30 µg of sample protein, diluted in 20 µl double distilled water (dd H_2O) and sample buffer (40 % glycerine, 10 % mercaptoethanol, 0.04 % bromphenol blue, and 250 mM Tris-HCl, pH 6.75) is denatured at 90 °C for 5 min and inserted into the stacking gel wells. Electrophoresis is run at a constant voltage of 150 V until the dye front reaches the bottom of the resolving gel (approximately 2 hours) with a running buffer containing 25 mM Tris, 195 mM glycine, 2 mM EDTA, and 0.1 % SDS. Proteins are then transferred from the gels onto polyvinylidene fluoride (PVDF) membranes in transfer buffer (192 mM glycine, 20% methanol, and 25 mM Tris) at a constant current of 250 mA for 75 min. Membranes can be stored at -20 °C until further use.

Prior to immunolabelling, the membranes are blocked in blocking buffer (tris-buffered saline (TBS) with 1% BSA) for 1 hour. The following primary antisera are used: mouse-monoclonal anti-vinculin (7F9, Santa Cruz Biotechnology, 1:1000), mouse monoclonal anti-tropomyosin (CH1-s, DSHB, 1:500), mouse monoclonal anti-α-tubulin (12G10, DSHB, 1:500), mouse monoclonal anti-GAPDH (6C5, Santa Cruz Biotechnology, 1:1500). HRP-conjugated polyclonal goat anti-mouse immunoglobulins (Dako, 1:10.000) are applied as secondary antibodies. All primary and secondary antibodies are diluted in blocking buffer and incubated for 1 h. After each antibody application, membranes should be extensively rinsed and washed (3 × 10 min) in TBS. Visualization of antibody binding is enabled by application of chemiluminescence substrate (Roti®-Lumin plus, Carl Roth) and photographed using a digital gel documentation system (Fusion X, Vilber).

4) Band documentation and interpretation:

Protein band intensities are measured using ImageJ software (ImageJ 1.45s, Java 1.6.0_20). Signals of the native bands with an intensity < 1% can be considered as background and thus as no band. Alterations of band patterns, such as the disappearance of a native band or appearance of additional bands, are considered as degradation events.
